# Toll-Like Receptors as a Therapeutic Target in the Era of Immunotherapies

**DOI:** 10.3389/fcell.2021.756315

**Published:** 2021-10-04

**Authors:** Mariya Farooq, Maria Batool, Moon Suk Kim, Sangdun Choi

**Affiliations:** ^1^Department of Molecular Science and Technology, Ajou University, Suwon, South Korea; ^2^S&K Therapeutics, Suwon, South Korea

**Keywords:** Toll-like receptor, TLR-based immunotherapies, cancer, SARS-CoV-2, infection, autoimmune disorder

## Abstract

Toll-like receptors (TLRs) are the pattern recognition receptors, which are activated by foreign and host molecules in order to initiate the immune response. They play a crucial role in the regulation of innate immunity, and several studies have shown their importance in bacterial, viral, and fungal infections, autoimmune diseases, and cancers. The consensus view from an immunological perspective is that TLR agonists can serve either as a possible therapeutic agent or as a vaccine adjuvant toward cancers or infectious diseases and that TLR inhibitors may be a promising approach to the treatment of autoimmune diseases, some cancers, bacterial, and viral infections. These notions are based on the fact that TLR agonists stimulate the secretion of proinflammatory cytokines and in general, the development of proinflammatory responses. Some of the TLR-based inhibitory agents have shown to be efficacious in preclinical models and have now entered clinical trials. Therefore, TLRs seem to hold the potential to serve as a perfect target in the era of immunotherapies. We offer a perspective on TLR-based therapeutics that sheds light on their usefulness and on combination therapies. We also highlight various therapeutics that are in the discovery phase or in clinical trials.

## Introduction

Infectious diseases and immune disorders have presented a substantial impediment to the growth, stability, and progress of all civilizations for centuries. Efforts to prevent or eradicate such diseases are arguably the most significant benefit that society has reaped from the contemporary era of biomedical sciences. For combatting various disorders and infections, it is crucial to gain a deeper understanding of how immune responses are triggered and modulated. In this regard, Toll-like receptors (TLRs) have emerged as a major topic of biomedical studies, because this family of proteins acts as one of the early determinants of immune activation (Fitzgerald and Kagan, 2020).

Toll-like receptors are type I transmembrane proteins containing conserved motifs known as the leucine-rich repeat and Toll/interleukin-1 receptor (TIR) domain (Nie et al., 2018). TLRs fall under the category of pattern-recognition receptors (PRRs) of the host defense system. PRRs recognize specific molecular patterns in microbes known as microbe-associated molecular patterns (MAMPs), pathogen-associated molecular patterns (PAMPs) as well as molecules released by a damaged cell of the host, which are known as damage-associated molecular patterns (DAMPs) (Vijay, 2018). Thus, PRRs participate in the development of an immune response against pathogens and help with the self-healing process of the cell. The other types of PRRs include Nod-like receptors, C-type lectin receptors, and RIG-like receptors (Takeuchi and Akira, 2010). TLRs were first identified in *Drosophila* embryos. Further studies have revealed that these receptors are involved in the development of antifungal responses in adult flies. Later, it was discovered that TLRs are also present in mammals. This finding has led to an understanding of a crucial part of the mechanism behind innate-immune-response development (Takeda and Akira, 2015).

Toll-like receptor recognition of microbial invaders activates a downstream signaling cascade that produces secreted cytokines and chemokines, which then activates both the innate and adaptive immune responses to clean infections (Shi et al., 2016). TLRs are present both on immune cells, including monocytes, macrophages, dendritic cells, neutrophils, B cells, T cells, mast cells, natural killer cells, and on non-immune cells, including fibroblasts, epithelial cells, astrocytes, keratinocytes, and platelets (Ospelt and Gay, 2010; Cognasse et al., 2015; Aletaha et al., 2017). TLRs are also presented by various tumor cells and their activation can promote or reduce the tumor growth. High level of TLRs has been found to be associated in disease aggressiveness, poor treatment outcome and development of therapeutic resistance in the cancer cells. Similarly in case of viral diseases, both TLR agonists and antagonists are used for the development of anti-viral state in the body (Muccioli and Benencia, 2014). Bacterial and autoimmune diseases are being treated with the help of TLR antagonists (Knapp, 2010; Hosseini et al., 2015). We discuss the role of TLRs in infectious diseases, cancers, and autoimmune diseases and therapeutic regimens which involve immune system modulation. Moreover, this review highlights the challenges in the discovery of TLR-based immunotherapies.

## Toll-Like Receptor Family: Phylogeny and Structure

Structurally, TLRs are remarkably conserved between humans and mice. To date, 13 TLRs have been identified. Humans have 11, whereas murine species have 13 TLRs (Yang et al., 2017). Humans do not have TLRs 11, 12, and 13, while some other species possess these TLRs. TLR10 is not expressed and TLR8 is not functional in mice (Huang et al., 2008). TLRs are homodimeric or heterodimeric transmembrane proteins that are expressed on the cell surface (TLRs 1, 2, 5, and 6), endosomal surface (TLRs 3, 7, 8, and 9), or both (TLR4) (Shekarian et al., 2017).

On the basis of phylogenetic analysis, human TLRs can be categorized into three subfamilies. The first subfamily consists of TLRs 1, 2, 6, and 10. The second subfamily is composed of TLRs 4 and 5, whereas the third one consists of TLRs 3, 7, 8, and 9. TLR subfamilies are classified based on the cellular localization. Subfamilies 1 and 2 are localized on the cell membrane. However, the subfamily 3 is localized in endoplasmic reticulum, which is trafficked to endosome upon activation (Lai et al., 2017).

Domain organization is similar among all TLRs because each TLR consists of a type 1 transmembrane protein. The ectodomain is composed of 16–28 leucine-rich repeats, while the conserved cytosolic signaling domain is known as the TIR domain, which activates TLR-specific adaptor proteins (Braunstein et al., 2018). Ectodomain leucine-rich repeats mediate the recognition of PAMPs (Kawai and Akira, 2011). The ectodomain is sandwiched between the N- and C-terminal domains. Both domains are joined by a single transmembrane helix (Matsushima et al., 2007). The TIR domain is present in the C-terminal region of mammalian TLRs. It is an important molecule in downstream signaling of the immune cascade. It recruits signaling adaptor proteins that are responsible for further downstream signal transduction necessary for an appropriate innate immune response (Ve et al., 2017).

## The Toll-Like Receptor Signaling Pathway

Upon stimulation, TLRs act via different signaling mechanisms, which induce the synthesis of various cytokines (Fitzgerald and Kagan, 2020). TLR signaling is initiated by the interaction between a TLR and ligand, leading to receptor dimerization. TIR domain–containing adaptor proteins (TIRAPs) are recruited by the TIR domain. There are five TLR adaptor proteins: MyD88 (myeloid differentiation primary-response gene 88), MAL (MyD88 adaptor-like protein), TRIF (TIR domain-containing adaptor protein inducing interferon-β (IFN-β), and TRAM (TRIF-related adaptor molecule) (O’Neill and Bowie, 2007).

Toll-Like receptors function in either MyD88-dependent or MyD88-independent pathways. Upon TLR activation, MyD88 and TRIF form myddosomes and triffosomes, respectively and initiate further signaling by recruiting kinases and downstream signaling molecules (Gay, 2019). In case of myddosome formation, IL-1R-associated kinases 1/4 (IRAK1/4) associates with TNF receptor–associated factor 6 (TRAF6), TRAF6 promotes polyubiquitination of both TRAF6 itself and the TAK1 protein kinase complex. TAK1 then activates two different pathways that lead to activation of the IKK complex-NF-κB pathway and -MAPK pathway. The IKK complex is composed of the catalytic subunits IKKα, IKKβ, and IKKγ (Israël, 2010). TAK1 activates IKKβ. The IKK complex phosphorylates the NF-κB inhibitory protein IκBα, which undergoes proteasome degradation, allowing NF-κB to translocate into the nucleus to induce proinflammatory gene expression. TAK1 activation prompt activation of mitogen-activated protein kinases (MAPK) which leads to recruitment of cyclic AMP-responsive element-binding protein (CREB), and activator protein 1 (AP-1) family transcription factors or stabilization of mRNA to regulate inflammatory responses (Kawasaki and Kawai, 2014; Achek et al., 2016). TLR3, which is triggered by double-stranded RNA, recruits the adaptor protein TRIF, which activates TRAF3 and IRF3. These two events induce type 1 and type 3 interferon secretion. Interferons are very important for efficient generation of antiviral responses. In TLR7, TLR8, and TLR9 signaling cascades, MyD88 activation leads to expression of genes of proinflammatory cytokines and type I and type III IFN through TRAF6 and interferon regulatory transcription factor (IRF) and NF-κB activation (Takeuchi and Akira, 2007; Lester and Li, 2014). TLRs also direct the adaptive immune response toward the T helper 1 phenotype by causing dendritic cells and macrophages to produce IL-12. In this way, a TLR(s) not only initiates an immune response against a pathogen, such as a bacterium, virus, or fungus, but also serves as a bridge between innate and adaptive immune systems (Huang et al., 2008).

**FIGURE 1:**
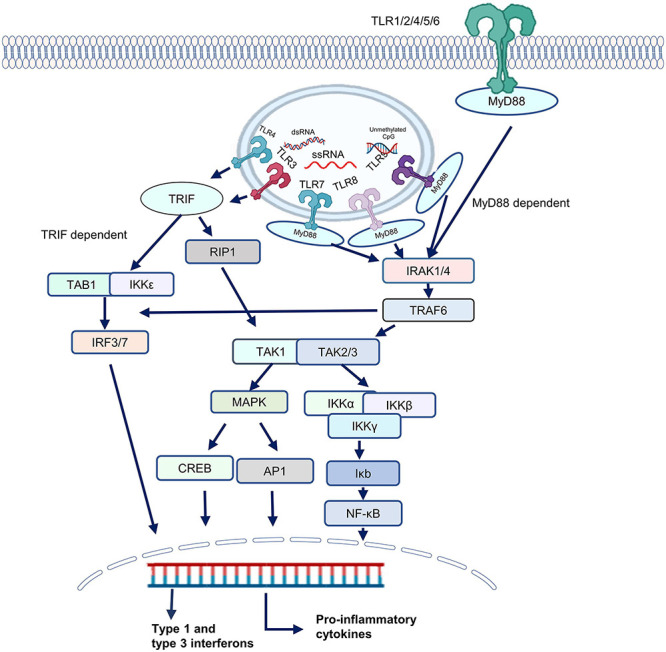
The Toll-like receptor (TLR) signaling pathway. TLRs are categorized into two groups depending upon whether they utilize a MyD88-dependent pathway or a TRIF-dependent pathway. TLRs 1,2, and 5–9 transmit signals through a MyD88-dependent pathway, whereas TLR3 signaling is mediated by a MyD88-independent pathway. By contrast, TLR4 can signal through MyD88-dependent and MyD88-independent pathways. In case of MyD88-dependent signaling, IL-1R-associated kinases 1/4 (IRAK1/4) recruit TNF receptor–associated factor 6 (TRAF6). TRAF6 can also stimulate interferon signaling by recruitment of IRFs. TRAF6 activates TAK1 which activates two distinct pathways, one of which leads to the activation of the IKK complex-NF-κB pathway and the other to the activation of the mitogen activated protein kinases (MAPKs). The catalytic subunits IKKα, IKKβ, and IKKγ make up the IKK complex. IKKβ is also activated by TAK1. The IKK complex phosphorylates the NF-κB inhibitory protein IκBα, which is then degraded by the proteasome, allowing NF-κB to enter the nucleus. TAK1 activation triggers the activation of MAPK family members leads to the activation of transcription factors such cyclic AMP-responsive element-binding protein (CREB), and activator protein 1 (AP-1). CREB, AP-1 and NF-κB then stimulate proinflammatory mediators and interferons. However, in case of TRIF-dependent signaling, IRF3 and IRF7 are recruited which lead to secretion of type 1 and type 3 interferon.

Toll-like receptor 4, however, is unique in terms of MyD88 signaling. TLR4 utilizes MyD88-dependent and TRIF-dependent pathways for signal transduction upon engagement by PAMP as presented in Figure 1 (O’Neill et al., 2013). In resting state, TLR4 is present on the plasma membrane and Golgi apparatus. Myeloid Differentiation factor 2 (MD-2), a secreted glycoprotein also known as LY96, is crucial for the translocation of TLR4 from Golgi apparatus to plasma membrane. Upon activation by LPS, TLR4 activates NF-κB via MAL and MyD88 complex formation. Then TLR4 translocate to endosome in a clathrin and dynamin dependent manner and binds to TRAM and TRIF, which results in the activation of IRF3 pathway. Thus, TLR4 activation is induced by LPS through TRIF and MyD88 pathways. However, transport to endosome limits MyD88 activity and enhances TRIF activity (McGettrick and O’Neill, 2010; Cheng et al., 2015). When LPS interacts with TLR4, different cofactors including MD2, LPS binding protein (LBP), and CD14 are involved in the activation of TLR4. CD14 recognizes the complex of TLR4 and LBP, CD14 binds to LBP and is involved in delivering the LPS-LBP complex to the TLR4-MD2 complex, localized on the plasma membrane (Yang et al., 2016).

## Toll-Like Receptors in Bacterial and Fungal Diseases

### Toll-Like Receptors Activation by Bacteria

Bacterial infections have always been a havoc for humanity and the effect has been amplified due to the multi-drug resistant bacterial strains. Infections caused by methicillin resistant *Staphylococcus aureus, Pseudomonas aeruginosa, Acinetobacter* species, and other pan resistant bacteria are still substantial burden to healthcare setups (Christou, 2011). It is speculated that the cell wall components of bacteria are recognized by the cell surface TLRs whereas the genomic components are recognized by the endosomal TLRs (Wagner, 2004). Activation of TLRs through bacterial ligands lead to development of antimicrobial response by activating production of pro-inflammatory cytokines as well as chemokines (Casilag et al., 2020). Among the TLRs found in mammals, TLR2, TLR4, TLR5, and TLR9 are reported to be stimulated by bacterial ligands. There is a panel of bacterial ligands which activate TLR2. It has been reported that Diacyl lipopeptides, (MALP-2/FSL-1) lipomannan, lipoarabinomannan, and lipoprotein from *Mycoplasma*, heat-labile enterotoxins from *Escherichia coli* and *Vibrio cholerae*, lipoteichoic acid from gram-positive bacteria, peptidoglycan from *Staphylococcus*, triacyl lipopeptides from bacteria, and porins from *Neisseria, Salmonella, and Shigella* serve as TLR2 microbial ligands (de Oliviera Nascimento et al., 2012).TLR4 recognizes LPS which is a component of gram-negative bacteria and leads to production of inflammatory cytokines, such as TNF-α, IL6, chemokines monocyte chemoattractant protein-1 (MCP-1), and C-X-C motif chemokine 10 (CXCL10) (Park and Lee, 2013; Yu et al., 2021). It has been reported that CD14 and MD-2 mediates the interaction between LPS and TLR4 as no direct interaction between LPS and TLR4 has been reported (Kawai and Akira, 2009; Płóciennikowska et al., 2015).

### Utilization of Bacterial Components as Vaccine Adjuvants and Therapeutic Regimens

The fact that TLRs are activated by bacterial cellular components points out toward the possibility of utilizing these ligands as vaccine adjuvants. Owing to potential to stimulate the appropriate immune response, various bacterial proteins have been proposed to be used as adjuvants against bacterial diseases (Kumar et al., 2019). Casilag et al. (2021) reported that for *Streptococcus pneumoniae* infection, outcome can be imporved by using the antibiotic in combination with TLR4 agonist. When amoxicillin was administered with monophosphoryl lipid A, it was observed that the antibiotic worked synergistically with the TLR agonist (Casilag et al., 2021). As bacterial DNA contains unmethylated CpG motifs, it activates TLR9 when the bacteria is internalized in the endosomal compartment (Gäbele et al., 2008). It has been reported that TLR9 served as a crucial receptor involved in the induction of type 1 interferons when infected with *Staphylococcus aureus* (Parker and Prince, 2012).

Multiple TLR4 antagonists have been proposed as therapeutic regimen against septic shock (Nijland et al., 2014). Eritoran is a synthetic lipid A molecule which inhibits the interaction between LPS and TLR4-MD2 (Opal et al., 2013; Kuzmich et al., 2017). It was tested in phase III trials, where it failed to show any improvement in the experimental group as compared to the control group (NCT00334828). Oversights in research design, patient population variances, improved patient care practices, and mixed bacterial infections are among the possible causes for Eritoran’s failure (Anwar et al., 2019). TAK-242, a well-known inhibitor of TLR4, could neither show any reduction in the cytokine levels nor any improvement in the patients when tested in clinical trials phase III (Gabarin et al., 2021). Strategy of inhibition of pro-inflammatory cytokines like IL6, TNF-α, and IL-1β has also failed against sepsis in clinical trials (Barratt-Due et al., 2017).

Nucleic acids released from the bacteria, also known MAMPs, are involved in the TLR activation as well. As bacterial DNA is capable of activating TLR9, its inhibitors hold potential to serve as a drug in various diseases (Savva and Roger, 2013). Chloroquine has been reported to downregulate TLR9 via NF-K B pathway (Zhang et al., 2015). Another compound curcumin, which is polyphenol in nature, has been reported to improve the septic acute kidney injury in Sprague-Dawley (SD) rats by downregulation of TLR9 and consequently, its downstream regulators including MyD88, IRF5, and IRF7 and hence inflammatory factors and organ injury markers (Li et al., 2021).

## Fungal Diseases and Toll-Like Receptors

Out of estimated 5 million fungal species present on earth, less than 100 are reported to be pathogenic which include *Aspergillus fumigatus*, *Cryptococcus neoformans*, and more recently *Cryptococcus gatii*, *Histoplasma capsulatum*, *Coccidiosis posadasii*, *Pneumocystis jirovecii*, and the commensal *P. jiroveci* or the *Candida* spp. Yet the disease burden caused by the fungal infections is substantial (Lionakis and Levitz, 2018). Currently, a very few antifungals have been discovered (Almeida et al., 2019). Fungi and their cellular components, particularly cell wall components, are recognized by various TLRs including TLR2, TLR6, TLR4, and TLR9 (Netea et al., 2007; Romani, 2011). Various strategies are utilized by fungi in order to evade the recognition by immune system, which includes sensing by TLRs. They can undergo cell wall remodeling and can form large cellular structures which can hamper phagocytosis, ultimately inhibiting the further immune response. It has been reported that DNA from *Cryptococcus neoformans* can downregulate TLR9 activation (Bourgeois and Kuchler, 2012). Van Der Graaf et al. (2005) reported that *Candida* blastoconidia lead to secretion of IFN-γ whereas *Candida* hyphae lead to production of proinflammatory cytokines, pointing toward a possible mechanism of immune evasion due through phenotypic switching.

## Toll-Like Receptors and Viral Infections

### Toll-Like Receptor Activation by Viral Proteins

Viruses present unique signatures to the immune system through nucleic acids. It has been reported that viruses activate the immune system, thereby starting IFN production to combat the viral infection. Nevertheless, after the discovery of TLRs, viral interactions with the innate immune system have been revised. It was found that TLRs recognize a virus, thus activating the immune system through an IFN-α or -β-dependent or -independent mechanism (Thompson and Iwasaki, 2008).

TLRs 3, 7, and 8 recognize viral RNA components, in contrast to other TLRs, which are located in the plasma membrane. TLRs 3, 7, and 8 are located in intracellular compartments and are triggered in late endosomal-lysosomal stages (Takeuchi and Akira, 2007). In the viral life cycle, enveloped viruses enter the cells by interacting with the receptors or attachment factors, which helps them to be internalized into the cells via a variety of endocytic processes. Examples of endocytic processes include clathrin-mediated endocytosis, micropinocytosis, clathrin- and caveolin-independent endocytosis. Some of the non-enveloped viruses enter the cells through clathrin- and caveolin-independent endocytosis (White and Whittaker, 2016). TLR3 recognizes double-stranded RNA, which is the genome in many virus families or an intermediate in the replication cycle of DNA viruses. TLR3 was also reported to be activated by virus-infected apoptotic cells (Thompson and Iwasaki, 2008).

Toll-like receptor 3 is located on the membrane of the endoplasmic reticulum in normal host cells. A double-stranded RNA molecule of at least 90 bp is recognized by endosomal TLR3 (Leonard et al., 2008). When a ligand stimulates the endosomal membrane, TLR3 dimerizes and is trafficked to the endosomal membrane, where it interacts with the antigen in a pH-dependent manner. The interaction between endoplasmic-reticulum protein UNC-93B and TLR3 is critical for the activation of the latter. Tyrosine phosphorylation on TLR3 is crucial for the recruitment of TIRAP and TRIF. In contrast, TLR7 signaling is MyD88-dependent and TRIF-independent and does not require the phosphorylation of tyrosine (Perales-Linares and Navas-Martin, 2013).

Toll-like receptor 7 and Toll-like receptor 8 have been found to recognize GU-rich and AU-rich single-stranded RNA molecules. This process generates a MyD88-dependent immune response against various viruses, such as vesicular stomatitis virus, influenza virus, Japanese encephalitis virus, and other flaviviruses (Nazmi et al., 2014).

It has been reported that 70–80% of mammalian DNA is methylated in CpG motifs. On the other hand, TLR9 recognizes unmethylated CpG motifs in DNA (Li and Zhang, 2014). In this way, TLR9 differentiates between host DNA and viral or bacterial genomic DNA. TLR9 recognizes various viruses, including murine cytomegalovirus, herpes simplex virus 1 (HSV1), HSV2, adenoviruses, poxviruses, Kaposi’s sarcoma herpesvirus, and varicella zoster virus. Active replication of viruses is not required for TLR9 triggering. UV-inactivated virions are also capable of engaging TLR9 (Lester and Li, 2014).

### Toll-Like Receptor and SARS-CoV-2

In a recent study, it has been depicted in a ferret model that the injection of TLR2/6 agonist significantly reduced the level of viral RNA in nose and throat (Proud et al., 2021). Similarly, another study reported that SARS-CoV-2 envelop protein was able to upregulate the production of proinflammatory cytokines through TLR2-dependent signaling (Zheng et al., 2021). *In silico* studies, phylogenetic analysis and preliminary studies on the interaction between SARS-CoV-2 and human cells suggest that endosomal TLRs are also a possible route of entry for SARS-CoV-2 (Bezemer and Garssen, 2021; Gadanec et al., 2021; Salvi et al., 2021). M5049, a small-molecule inhibitor developed by Merck, is an antagonist of TLR7 and TLR8. Currently, it is undergoing clinical trials (NCT04448756). Pul-042 is an agonist of TLR2-TLR6 and TLR9. This agonist is used as a prophylactic drug against COVID-19 and is currently in clinical trials against reducing the infection rate and progression of COVID19 (NCT04313023). Imiquimod, which is preferentially TLR7 agonist also activates TLR8 weakly, is a similar kind of drug that can be employed for prophylaxis in such patients (Scho and Scho, 2008; Avcilar and Eken, 2020). Ruxolitinib is a small-molecule inhibitor of Janus kinase (JAK) is known to downregulate TNF-α, has been tested in clinical trials phase III (NCT04362137). Emapalumab and anakinra are monoclonal antibodies that antagonize IFN-γ and IL-1β, respectively, and are currently in clinical trials (NCT0432402). Tocilizumab and sarilumab are IL-6 inhibitors and belong to a class of monoclonal antibodies. Their effects on COVID-19 patients are being studied too (NCT04479358)(NCT04386239) (Choudhury and Mukherjee, 2020). Anakinra and Tocilizumab are also being used in combination against COVID-19 (NCT04412291).

**FIGURE 2:**
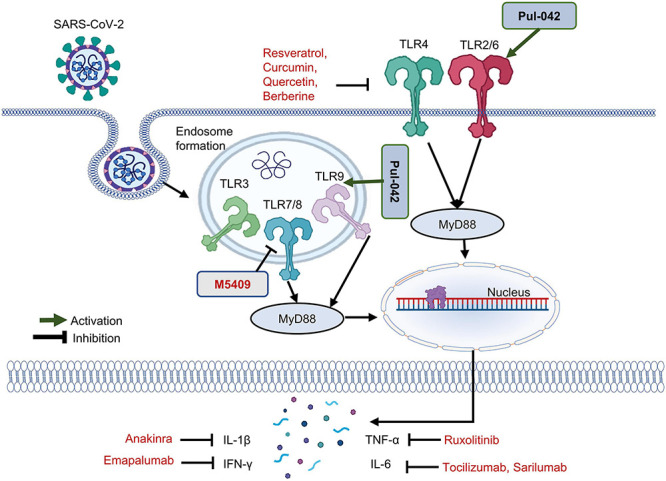
Toll-like receptor (TLR) triggering SARS-CoV-2 infection. SARS-CoV-2, in addition to its main receptor ACE-2, can enter the cell through interaction with TLRs. Currently, TLR4 antagonists including resveratrol, curcumin, quercetin, and berberine are in clinical trials. Pul-042 is a TLR2/6/9 agonist, which stimulates T-helper cells mediated cytokine and interferon secretion. To control a cytokine storm, anakinra, emapalumab, and ruxolitinib are being tested, which are monoclonal antibodies against IL-1β, IFN-γ, and TNF-α. Against IL-6, tocilizumab and sarilumab can be used. M5049, a TLR7/8 antagonist, is currently being tested in clinical trials against COVID.

It has been proved in an *in silico* study that the S1 subunit of the spike protein of SARS-CoV-2 strongly binds to TLR4 (Choudhury and Mukherjee, 2020). Some researchers proposed a model suggesting that the binding between SARS-CoV-2 and TLR4 enhances the expression of ACE-2 on the cell surface, thus facilitating virus entry (Aboudounya and Heads, 2021; Pantazi et al., 2021). Although there is not much evidence, TLR4 is also considered a possible TLR involved in the entry of SARS-CoV-2 into the cell. Multiple TLR4 agonists and antagonists are currently in clinical trials against SARS-CoV-2. Some of the TLR4 antagonists that are in clinical trials include resveratrol, curcumin, quercetin, and berberine (Kaushik et al., 2021; Figure 2).

### Viral Manipulation of Host Immune System

Virus recognition by the host immune system is not limited to endosomal components such as endosomal TLRs, STING receptors, and RLRs. Additionally, the viral components produced by the cell are recognized by TLRs located on the plasma membrane. Some investigators reported that microRNAs produced by human cytomegalovirus downregulate TLR2 expression and thus modulate the innate immune response (Landais et al., 2015). TLR2 can recognize human-cytomegalovirus glycoproteins. A large number of viral proteins are known to turn on TLR2, TLR4, and TLR6 (Boehme et al., 2006; Kawai and Akira, 2011). HCMV encoded microRNA miR-UL112-3p has been reported to downregulate the TLR2 expression in a similar fashion to TLR2 targeting siRNA. It was also reported that upon TLR2 stimulation, miR-UL112-3p downregulated the expression of multiple cytokines (Landais et al., 2015).

Vaccinia virus protein A46R contains a TIR domain that interacts with MyD88, TRIF, and TRIF-related adaptor molecules and downregulates TRIF-mediated IFN secretion (Stack et al., 2005). A46R is thought to be an immunomodulatory protein that targets MAL and MyD88 (Azar et al., 2020). Another vaccinia virus protein, A52R, inhibits multiple TLRs by targeting TRAF6 and IRAK2 (Harte et al., 2003). The discovery of these two proteins indicates that it is possible to use viral proteins as TLR ligands (Patel et al., 2014).

### Toll-Like Receptor-Based Immunotherapeutics to Combat Viral Infections

Toll-like receptors are employed as therapeutic targets as well as vaccine adjuvants in viral infections. FDA-approved antiviral drugs include many TLR agonists. Examples include monophosphoryl lipid A, which is a TLR4 agonist and is utilized as an adjuvant in vaccines against human papilloma virus and hepatitis B virus. Imiquimod (brand name Aldara^TM^) which is mainly TLR7 agonist, has been approved for human-papilloma-virus–induced genital and perianal warts. Flagellin (a TLR5 agonist) and Class B CpG (a TLR9 agonist) serve as adjuvants in influenza vaccines. Poly I:C, a TLR3 agonist, is used as an antiviral agent against influenza virus strains (Hedayat et al., 2011). Aside from FDA-approved drugs, many TLR ligands have been experimentally tested for the development of antiviral drugs, and some of them are in clinical trials (Patel et al., 2014).

### Immune Sensors for Viruses Other Than Toll-Like Receptors

Intracellular viral RNA is also recognized by another class of PRRs known as retinoic acid-inducible gene-I (RIG-I)-like receptors (RLRs) (Cao et al., 2019). RLRs are dsRNA binding proteins, that recognize RNA which is distinct from the host RNA based upon the molecular characteristics (Zhao and Karijolich, 2019). The three members of RLRs are RIG-1, MDA5 and MAVS. Both RIG-1 and MDA5 are localized in the cytosol. Both of them contain N-terminal tandem caspase-recruitment domains (CARDs) and helicase domains. When dsRNA binds to RIG-1 or MDA5, oligomerization of the RNA sensors occurs which leads to activation of MAVS. As a result, IRF3/7 along with NF-κB gets activated and Interferon production is induced (Uehata and Takeuchi, 2020).

RIG-1 is activated by RNA molecules with 5′ cap, di- or triphosphate groups attached, no ribose and 2′-O-methylation and having a duplex structure. These characteristics save the immune system from getting into trouble of self-recognition as most of these characteristics are present in viral genomes but absent in the host RNAs (Rehwinkel and Gack, 2020).

Activation of MDA5 is not understood completely yet. RNA and DNA viruses have been reported to activate MDA5. It has been proposed that when double stranded RNA viruses infect cells, it leads to accumulation of RNA inside the cells. The accumulation of viral RNA is responsible for the activation of MDA5 (Rehwinkel and Gack, 2020).

Stimulator of interferon genes (STING), also known as transmembrane protein 173 (TMEM173), is a 379 amino acid protein in humans and 378 amino acid protein in mouse cells. It is usually expressed the immune cells and is localized in the endoplasmic reticulum. STING pathway is activated by cyclic dinucleotides (CDNs), which are produced by bacteria after they infect the cells. CDNs are also synthesized by cellular synthase also known as cyclic GMP–AMP synthase (cGAS) (Ahn and Barber, 2019). cGAS is the DNA sensor which recognizes the viral, bacterial and host DNA. CDNs induce the STING which undergo confirmational changes upon activation. The N-terminal domain of STING is responsible for the homodimerization and cellular localization, whereas the C-terminal recruits the other signaling molecules. Once STING is activated, it is transported to the endoplasmic reticulum, then Golgi apparatus and finally to the endosome, through intracellular trafficking or autophagy. It ultimately leads to secretion of type 1 interferon, type III interferon and other proinflammatory genes (Li et al., 2020).

## Toll-Like Receptors and Cancers

Deidier was the first person (in the 18th century) to observe a positive correlation between a cure of cancer and infection (Cao et al., 2019). In the 19th century, William Coley upon detailed analysis of the history of the patients, found 47 cases from the hospital record which depicted the advantageous effects of fevers on malignancy. From his observation, he also inferred that a mixture of bacterial toxins from gram-positive and gram-negative bacteria had strong antitumor effects when injected in a person suffering from malignant cancer (Urban-Wojciuk et al., 2019). The first patient in which he observed this effect was suffering from an inoperable tumor in neck (Carlson et al., 2020). A mixture of killed *Streptococcus pyogenes* and *Serratia marcescens* was termed as Coley’s toxins, and it was later discovered by Shear et al. (1943) that the ingredient of Coley’s toxins responsible for the antitumor effect is lipopolysaccharide (LPS) (Hiraku et al., 2014). As LPS is the classic agonist for TLR4, so it is clear that in case of Coley’s toxin the activation of TLR4 through LPS lead to clearance of tumor efficiently (Rakoff-Nahoum and Medzhitov, 2009). This phenomenon of successful treatment of tumor through activation of TLR4 points to a promising mechanism of anticancer therapeutic strategies.

### Immune Modulation by Tumor Cells

Tumors are thought to be recognized by the host immune system, but aggressive cancers progress via modulation of this system. For cancer immunotherapy to be successful, it is important to overcome the immunosuppressive nature of cancer cells. A recent advancement of cancer immunotherapy is a phenomenon known as “*in situ* vaccination.” In the tumor microenvironment, this approach creates an immunostimulatory microenvironment similar to that of a tumor that has already been treated (Mao et al., 2020). Hammerich et al. (2019) reported that *in situ* vaccination can successfully treat indolent non-Hodgkin’s lymphoma. This kind of vaccine contains a TLR3 agonist along with a tyrosine kinase 3 ligand and is combined with radiotherapy. As a result, an anti-tumor CD8^+^ T-cell response is generated. Even in non-responsive patients, a population of PD1^+^CD8^+^ T cells is formed. In this context, untreated distant tumors become sensitive to PD1 treatment; the addition of PD1 treatment increases the therapeutic efficacy from 40% to 80% (Hammerich et al., 2019).

Apoptotic cells trigger the inflammatory pathway via MyD88-mediated TLR activation. This process is known as a “sterile inflammatory response.” Cells undergoing apoptosis release molecules that serve as ligands for TLRs. In cells that are capable of switching on the MyD88-mediated TLR pathway, these molecules from dying cells cause NF-κB activation (Albensi, 2019). NF-κB in turn regulates the transcription of more than 100 genes involved in inflammation, including *IL6*, *TNFA*, and *IL1B* (Pradere et al., 2014). The activation of NF-κB leads to the production of proinflammatory cytokines, ultimately resulting in the inhibition of apoptosis and a progrowth microenvironment. Apoptosis inhibition has been proposed to be the reason for the chemoresistance of cancer. In this regard, TLRs serve as a double-edged sword that can turn against the body by initiating pathways that ultimately enhance cancer progression and induce chemoresistance (Chen et al., 2008).

### Controversial Role of Toll-Like Receptors in Cancer

In addition to immune cells, various types of tumor cells also express TLRs. It was demonstrated that the inhibition or promotion of tumor progression is regulated by different TLRs in tumor cells (Huang et al., 2018). The same research group reported that TLR9 is overexpressed in human tumor cells. However, in mice TLR4 is overexpressed in tumor cells (Huang et al., 2008). The role of TLRs in cancer has been considered controversial. Some investigators nominated TLR agonists as the best frenemy of cancer immunotherapy (Kaczanowska et al., 2013).

Nevertheless, TLRs are a double-edged sword in cancer progression. TLR4/MyD88 pathway has been found to be upregulated in breast cancer cell line with high invasion rate as compared to the breast cancer cell line with lower invasion rate. It was also suggested that TLR4/MyD88 levels could serves as a useful prognostic biomarker for breast cancer patients (Wu et al., 2018). Szajnik et al. (2009) suggested that in ovarian cancer, LPS mediated upregulated TLR4/MyD88 might assist cancer cells in progression and development of chemoresistance ultimately aiding them in immune evasion. However, TLR4 agonists have been tested for the development of antitumor response against bladder cancer (Moghadam and Nowroozi, 2019).

There are multiple combinatorial immunotherapies of cancer that involve TLR agonists and antagonists. In 2013, the Science magazine named cancer immunotherapy a “Breakthrough of the year.” Currently, there are a number of TLR agonists that are FDA-approved drugs and are being used for cancer immunotherapy. The predominantly TLR7 ligand imiquimod (also weakly activates TLR8) and a TLR9 ligand (a CpG oligo) have shown promising results in cancer treatment (Murad and Clay, 2009; Huang et al., 2018). The BCG vaccine involves TLR1-TLR2, TLR2-TLR6, TLR4, and TLR9 agonists and is reported to be effective against urothelial tumors (Kaczanowska et al., 2013; Larue et al., 2013). It is currently a standard of care for localized bladder cancer. AS15 is a TLR4 agonist and is employed as a cancer vaccine adjuvant (Braunstein et al., 2018). Several TLR agonists which are currently in clinical trials as cancer immunotherapies are mentioned in the Table 1.

**TABLE 1:** TLR-based ligands in clinical trials.

**Name**	**TLR being targeted**	**Agonist/antagonist**	**Disease**	**Identifier number**	**Last updated**	**Status**	**Phase**
**Cancers**							
CBLB612	TLR2	Agonist	Breast cancer	NCT02778763	July 20, 2016	Completed	Phase 2
Hespecta	TLR2	Agonist	Head and neck cancer	NCT02821494	February 21, 2021	Completed	Phase 2
Poly-ICLC	TLR3	Agonist	Low grade lymphoma	NCT01976585	November 19, 2020	Recruiting	Phase1/2
Poly-ICLC	TLR3	Agonist	Advanced Cutaneous T Cell Lymphoma	NCT02061449	December 24, 2018	Terminated	Phase 1
GLA-SE	TLR4	Agonist	Melanoma	NCT02320305	January 14, 2020	Completed	Early phase 1
GLA-SE	TLR4	Agonist	Soft tissue sarcoma	NCT02180698	November 6, 2019	Completed	Phase 1
GLA-SE	TLR4	Agonist	Follicular Non-Hodgkin’s Lymphoma	NCT02501473	September 9, 2020	Terminated	Phase 1/2
ONT-10	TLR4	Agonist	Ovarian or breast cancer	NCT02270372	May 17, 2018	Completed	Phase 1
Mobilan	TLR5	Agonist	Prostate Cancer	NCT02844699	September 20, 2017	Unknown	Phase 1/2
Entolimod	TLR5	Agonist	Metastatic solid tumors	NCT01527136	January 12, 2016	Completed	Phase 1
Entolimod	TLR5	Agonist	Squamous Cell Head and Neck Cancer	NCT01728480	December 11, 2013	Withdrawn	Phase 1
Imiquimod	TLR7	Agonist	Breast cancer	NCT01421017	December 24, 2018	Completed	Phase1/2
Resiquimod	TLR7/8	Agonist	Tumors	NCT00821652	January 8, 2015	Completed	Phase 1
IMO-8400	TLR7/8/9	Antagonist	Diffuse Large B Cell Lymphoma	NCT02252146	December 12, 2017	Completed	Phase 1/2
VTX-2337	TLR8	Agonist	Metastatic Squamous Cell Carcinoma of the Head and Neck	NCT01836029	October 29, 2019	Completed	Phase 2
Eritoran (E5564)	TLR4	Antagonist	Leukemia	NCT00756912	July 11, 2014	Terminated	Phase 1
**Viral infections**							
SD-101	TLR9	Agonist	HCV	NCT00823862	April 16, 2019	Completed	Phase 1
Resiquimod	TLR7/8	Agonist	Influenza	NCT01737580	August 28, 2013	Completed	Phase 1
Lefitolimod	TLR9	Agonist	HIV	NCT04357821	November 17, 2020	Recruiting	Phase1/2
Vesatolimod	TLR7	Agonist	CHB	NCT02166047	October 14, 2020	Completed	Phase 2
GS-9620	TLR9	Agonist	HBV	NCT01590654	December 20, 2013	Completed	Phase 1
CpG 1018	TLR9	Agonist	HIV	NCT04177355	September 2, 2021	Recruiting	Phase 1
VAX125	TLR5	Agonist	Influenza	NCT00966238	October 2, 2014	Completed	Phase 2
Poly-ICLC	TLR3	Agonist	HIV	NCT02071095	March 13, 2018	Completed	Phase 1/2
Poly-ICLC	TLR3	Agonist	HIV	NCT01127464	May 8, 2014	Completed	Phase 1
Imiquimod	TLR7	Agonist	Influenza	NCT02103023	December 5, 2014	Completed	Phase 3
Imiquimod	TLR7	Agonist	HPV	NCT00941811	June 16, 2015	Completed	Phase 2
Vesatolimod	TLR7	Agonist	HBV	NCT02166047	October 14, 2020	Completed	Phase 2
GS-9620	TLR7	Agonist	HBV	NCT01590654	December 20, 2013	Completed	Phase 1
RO7020531	TLR7	Agonist	CHB	NCT02956850	July 7, 2021	Completed	Phase 1
MGN1703	TLR9	Agonist	HIV	NCT02443935	June 29, 2017	Completed	Phase 1/2
CpG 7909	TLR9	Agonist	HIV	NCT00562939	January 21, 2009	Completed	Phase 1/2
IMO-2125	TLR9	Agonist	HCV	NCT00728936	February 15, 2019	Completed	Phase 1
M5049	TLR7/8	Antagonist	SARS-CoV-2	NCT04448756	August 5, 2021	Active, not recruiting	Phase 2
Pul-042	TLR2/6/9	Agonist	SARS-CoV-2	NCT04313023	September 2, 2021	Completed	Phase 2
**Autoimmune diseases and bacterial diseases**							
TAK-242	TLR4	Antagonist	Sepsis	NCT00143611	February 2, 2012	Completed	Phase 3
TAK-242	TLR4	Antagonist	Sepsis	NCT00633477	January 18, 2013	Terminated	Phase 3
TAK-242	TLR4	Antagonist	Alcoholic hepatitis	NCT04620148	August 2, 2021	Recruiting	Phase 2
IMO-3100	TLR7/9	Antagonist	Psoriasis	NCT01622348	January 9, 2018	Completed	Phase 2
IMO-8400	TLR7/8/9	Antagonist	Psoriasis	NCT01899729	August 10, 2020	Completed	Phase 2
LPS	TLR4	Agonist	Sepsis	NCT02554630	February 2, 2021	Recruiting	NA
Eritoran (E5564)	TLR4	Antagonist	Sepsis	NCT00334828	July 25, 2017	Completed	Phase 3
Eritoran (E5564)	TLR4	Antagonist	Severe sepsis	NCT00046072	December 12, 2005	Completed	Phase 1
Eritoran (E5564)	TLR4	Antagonist	Obese and Type 2 Diabetic Subjects	NCT02267317	March 4, 2020	Terminated	Phase 2
JKB-122	TLR4	Antagonist	Autoimmune hepatitis	NCT02556372	July 21, 2020	Completed	Phase 2

*HCV, Hepatitis C Virus; HBV, Hepatitis B Virus; HPV, Human Papilloma Virus; HIV, Human Immunodeficiency Virus; CHB, Chronic Hepatitis B; NA, not available.*

## Toll-Like Receptors and Autoimmune Diseases

The immune system, to differentiate self-antigens from foreign antigens, develops tolerance to self-antigens. On the other hand, if the immune system loses the ability to distinguish self- and non-self-antigens, an autoimmune disease develops. More than 80 types of autoimmune diseases have been documented so far (Smatti et al., 2019). TLR signal transduction is necessary for inducing the secretion of proinflammatory cytokines and hence for initiating an adaptive immune response. TLRs serve as a link between innate and adaptive immune responses. Therefore, dysregulation of TLRs is believed to be involved in the pathogenesis of autoimmunity (Hosseini et al., 2015). TLRs 7 and 9 have been found to be associated with systemic lupus erythematosus in humans and in mouse models. Activated TLRs lead to the upregulation of proinflammatory cytokines and interferon response from innate immune cells (Liu et al., 2017; El-Zayat et al., 2019).

Autoreactive B cells and overactive T cells are considered the main drivers of autoimmunity. Autoantigens interact with dendritic cells, which stimulate B cells, thereby resulting in the upregulation of autoantibodies and proinflammatory-cytokine production. In patients with systemic lupus erythematosus, TLR7 and TLR9 play an important part in the expansion of autoreactive B cells (Toubi and Vadasz, 2019).

### Role of Toll-Like Receptors in Psoriasis

Endosomal TLRs (TLRs 3, 7, 8, and 9) have been directly linked to the pathogenesis of multiple autoimmune diseases (Theofilopoulos et al., 2017). Psoriasis is a chronic autoimmune dermatosis with a prevalence of 2–3% in the world population. It is a systemic disease that can cause obesity, non-alcoholic fatty liver disease, cardiovascular disease, atherosclerosis, diabetes, hypertension, and inflammatory bowel disease (Korman, 2020). TLRs are also present on non-immune cells. Normally, keratinocytes express TLRs 1, 2, and 5. When cultured *in vitro*, they express TLRs 2–6 and 9. Activation of TLRs 2–4 results in the secretion of large amounts of proinflammatory cytokines, including TNF-α, IL-2, and a type I IFN (Rahmani and Rezaei, 2016). Heat shock proteins are reported to take part in the triggering of TLRs, thus increasing a proinflammatory-cytokine release, leading to inflammation in psoriasis (Sweeney et al., 2011).

### Therapeutic Interventions Targeting Toll-Like Receptors

There are several antibodies, including ixekizumab, adalimumab, and ustekinumab, that are currently used as drugs against psoriasis; they block the cytokines implicated in the development of psoriasis (Kuhn and Luger, 2010; Leonardi et al., 2011, 2019; Ren and Dao, 2013; Burness and McKeage, 2015; Zweegers et al., 2017). Several small-molecule inhibitors, including CPG-52364, IMO-8400, and IMO-3100, that target the activation of endosomal TLRs, are under clinical investigation against psoriasis. Some other small-molecule compounds have been tested in animal models as anti-inflammatory agents and found to effectively target the TLR signaling pathway too. Nonetheless, they have not been tested against psoriasis yet (Lai et al., 2017).

### Rheumatoid Arthritis

Rheumatoid arthritis is a chronic autoimmune arthropathy characterized by progressive articular destruction and a loss of function (Firestein and McInnes, 2017). The clinically symptomatic phase is preceded by pre-RA, which may be a few months to years long, and features increased levels of autoantibodies and cytokines in the blood. Clinical onset of the disease involves synovitis and the comorbidities that may affect metabolism, bones, the vascular system, and psychological state (McInnes and Schett, 2017).

Synovium is responsible for the maintenance of cartilage nutrition and joint function in healthy individuals. The synovial lining, composed of fibroblast-like synoviocytes and macrophage-like synoviocytes, expands after RA onset. Upregulation of TLRs 2, 3, 4, and 7 in fibroblast-like synoviocytes drives the expansion of the intimal lining. Overexpression of these TLRs causes upregulation of IL-6 and MMP3 (Arleevskaya et al., 2019). TLR3 and TLR4 are overexpressed in human synovial fibroblasts (Goh and Midwood, 2012). Upregulated TLR4 also contributes to development of neuropathic pain in RA (Pierer et al., 2011). It has been reported that upon inhibition of TLR4, severity of the disease was significantly reduced along with the cytokine level in the mice model (Abdollahi-Roodsaz et al., 2007). TLR4 seems to be an emerging target for controlling persistent pain in RA (Bruno et al., 2018).

### Therapeutic Interventions

**FIGURE 3:**
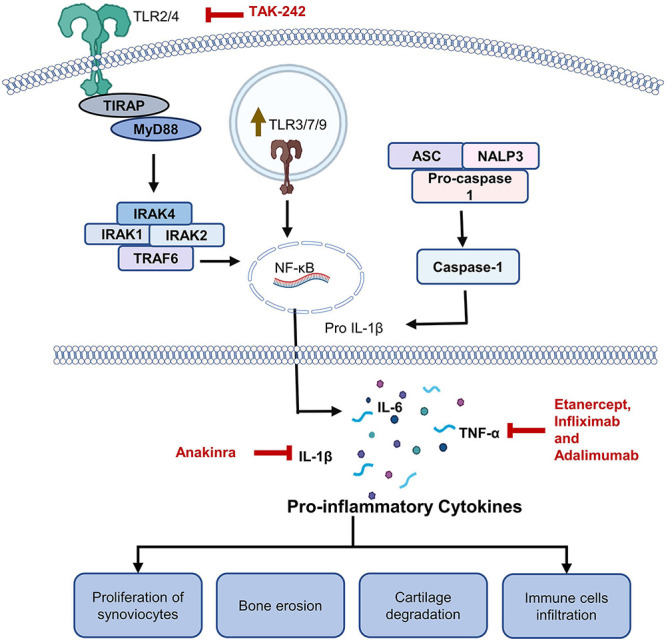
Roles of TLRs in rheumatoid arthritis (RA). Role of TLR2/4 has been extensively studied in RA. Engagement of a TLR by a DAMP promotes release of certain cytokines through MyD88 dependent pathway. Level of TNF-α, IL-6, and IL-1β have been found to be upregulated in RA patients. Upregulated cytokines lead to infiltration of immune cells, proliferation of synoviocytes ultimately leading to bone damage and cartilage degradation. Multiple monoclonal antibodies against TNF-α including etanercept, infliximab and adalimumab are commonly used for RA management. Patients who do not respond to anti-TNF-α antibodies are treated with anti–IL-1β antibodies. TAK-242, which is a TLR4 inhibitor, is also being tested against RA.

Sacre et al. (2008) demonstrated that TNF-α is induced upon stimulation of TLRs 3 and 8 in an RA model based on synovial cultures, thus pointing to their importance in RA therapeutic options. Cytokine levels in RA-synovial membranes are reduced upon inhibition of MyD88 and TIRAP, which are adaptor proteins for TLR2 and TLR4. It can be concluded that TLR2 and TLR4 are important in RA pathogenesis (Goh and Midwood, 2012). It was shown that TLRs 3 and 4 are overexpressed in synovial tissues at the early stages of RA, thereby causing chronic inflammation and dysfunction of joints (Ospelt et al., 2008). TAK-242, a specific inhibitor of TLR4-mediated signaling, has good potential for controlling the progression of RA, as illustrated in Figure 3. Samarpita et al. (2020) performed *in vitro* experiment using human rheumatoid fibroblast-like synoviocyte (FLS) line MH7A or primary human FLS and in an adjuvant-induced arthritis (AIA) rat model. TAK-242 was found to be effective in downregulating IL-6, IL-8, MMP-1, and VEGF level in LPS stimulated cells. It also hindered the translocation of NF-κB in nucleus. In rat model, TAK-242 at 5mg.kg alleviated the symptoms of RA and normalized the serum level of IL-6 and VEGF (Ono et al., 2020; Samarpita et al., 2020).

NI-0101 is a humanized monoclonal antibody against TLR4, which was tested in clinical trial phase II against rheumatoid arthritis (NCT03241108). No significant response in the treated patients could be observed (Monnet et al., 2020). Some available modalities against RA include etanercept, infliximab, and adalimumab targeting TNF-α and are known to reduce inflammation (Zamora-Atenza et al., 2014). Anakinra is a monoclonal antibody that inhibits IL-1β and has corresponding adverse effects. It is administered subcutaneously to patients who do not respond to a TNF-α inhibitor (Baskar et al., 2016; Abbasi et al., 2019).

## Toll-Like Receptortlrs in Pain and Neurodegeneration

Role of TLRs has been studied in neurological pain and TLR2,3,4,5,7,8,9 are supposed to be involved in the development of neurological pain (Thakur et al., 2017; Barrat, 2018). It has been reported that presence of TLRs on the central nervous system cells plays a key role in the maintenance and sustenance of neurological pain (Shah and Choi, 2017). Microglia predominantly express TLR2 and TLR4 and in certain circumstances, astrocytes can also express TLR2 and TLR4. Jurga et al. (2016) suggested that by blocking TLR2/4, pain can be relieved in rat neuropathic pain model. He et al. (2020) explained the involvement of TLR7 in the neuropathic pain in dorsal root ganglia. He suggested that as a result of neuronal injury, activation of TLR7 leads to upregulation of NF-κB, which contributed to the development of neurological pain. This effect can be blocked by inhibiting TLR7 (He et al., 2020).

Neurodegeneration refers to the gradual damage and death of neurons, and it normally occurs as a result of neurodegenerative diseases which include Parkinson’s disease, diabetic neuropathy, Alzheimer’s disease (Simó et al., 2018). There are various pathological conditions which can serve as a contributing factor to neurodegeneration, some of them also point out toward involvement of TLRs. One hypothesis, known as endotoxin hypothesis suggests that endotoxins are the cause or contributing factor for neurodegeneration (Brown, 2019). Zhang et al. (2018) reported that injection of *Porphyromonas gingivalis* LPS in C57BL/6 mice lead to activation of astrocytes and microglia in the cerebral cortex and hippocampus. It leads to upregulation of proinflammatory cytokines and activation of TLR4/NF-κB pathway. The effects were significantly reduced by using TAK-242, which is an inhibitor of TLR4 (Zhang et al., 2018).

## Discussion

Toll-like receptors are a class of PRRs that play a crucial role in the development of an innate immune response. Upon their engagement by a PAMP or DAMP, TLR dimerization results in the activation of transcription factors driving the secretion of proinflammatory cytokines. Endosomal TLRs are strongly involved in the recognition of viral genomic material and thus help to mount an antiviral response. Additionally, it is possible that cell surface TLRs are also turned on by components of viruses other than the genome, namely, by proteins generated for the virus by the cell. As for SARS-CoV-2 infection, TLRs are still a subject of debate. Nonetheless, various TLR agonists and inhibitors are currently being tested in clinical trials against COVID-19. Other viral infections, including those caused by vesicular stomatitis virus, Japanese encephalitis virus, and many flaviviruses, are also being treated with TLR ligands.

Furthermore, TLRs are an important but tricky target when it comes to cancer treatment. They serve as a double-edged sword in cancers. As a result of MyD88 engagement by TLRs, cancer cells undergo apoptosis. As a consequence of the interaction of the molecules released from cells, cytokines are produced, and apoptosis is inhibited. The suppression of apoptosis creates a favorable environment for tumor growth. That is why TLRs have been regarded as the best frenemy in cancer immunotherapy. As for autoimmune diseases, TLRs are involved in successful treatments of various diseases, such as psoriasis and RA. In the era of multi-drug resistant and pan-resistant bacterial infections, TLR-based drugs seem to be a ray of light. As far as fungal diseases are concerned, data suggests that drugs targeting TLRs sound promising. Therefore, it has become crystal clear that TLRs can serve as a promising target for the development of immunotherapeutics against many types of diseases.

Most of the diseases which are contributing a lot toward the global burden of diseases can be treated successfully with the successful discovery of TLR-based therapeutics. TLR-based therapeutics discovery seems to be tough, but it is exciting, and it will open new avenues in terms of drug discovery and development and prevention for infectious diseases, as well as cancer, allergies, asthma, and autoimmune diseases, either directly or through the development of better vaccines.

## Author Contributions

MF and MB defined the scope of the manuscript and wrote it. MF, MB, and SC revised the manuscript. MK and SC did the project administration and funding acquisition. All authors read and approved the final manuscript.

## Conflict of Interest

MB and SC was employed by company S&K Therapeutics, South Korea. The remaining authors declare that the research was conducted in the absence of any commercial or financial relationships that could be construed as a potential conflict of interest.

## Publisher’s Note

All claims expressed in this article are solely those of the authors and do not necessarily represent those of their affiliated organizations, or those of the publisher, the editors and the reviewers. Any product that may be evaluated in this article, or claim that may be made by its manufacturer, is not guaranteed or endorsed by the publisher.

## References

[B1] Abbasi M. Mousavi M. J. Jamalzehi S. Alimohammadi R. Bezvan M. H. Mohammadi H. (2019). Strategies toward rheumatoid arthritis therapy; the old and the new. *J. Cell. Physiol.* 234 10018–10031. 10.1002/jcp.27860 30536757

[B2] Abdollahi-Roodsaz S. Joosten L. A. B. Roelofs M. F. Radstake T. R. D. J. Matera G. Popa C. (2007). Inhibition of toll-like receptor 4 breaks the inflammatory loop in autoimmune destructive arthritis. *Arthritis Rheum.* 56 2957–2967. 10.1002/ART.22848 17763416

[B3] Aboudounya M. M. Heads R. J. (2021). COVID-19 and toll-like receptor 4 (TLR4): SARS-CoV-2 may bind and activate TLR4 to increase ACE2 expression, facilitating entry and causing hyperinflammation. *Mediators Inflamm.* 2021:8874339. 10.1155/2021/8874339 33505220 PMC7811571

[B4] Achek A. Yesudhas D. Choi S. (2016). Toll-like receptors: promising therapeutic targets for inflammatory diseases. *Arch. Pharm. Res.* 39 1032–1049. 10.1007/s12272-016-0806-9 27515048

[B5] Ahn J. Barber G. N. (2019). STING signaling and host defense against microbial infection. *Exp. Mol. Med.* 51 1–10. 10.1038/s12276-019-0333-0 31827069 PMC6906460

[B6] Albensi B. C. (2019). What is nuclear factor Kappa B (NF-κB) doing in and to the mitochondrion? *Front. Cell Dev. Biol.* 7:154. 10.3389/FCELL.2019.00154 31448275 PMC6692429

[B7] Aletaha S. Haddad L. Roozbehkia M. Bigdeli R. Asgary V. Mahmoudi M. (2017). M2000 (β-D-mannuronic acid) as a novel antagonist for blocking the TLR2 and TLR4 downstream signalling pathway. *Scand. J. Immunol.* 85 122–129. 10.1111/sji.12519 27943385

[B8] Almeida F. Rodrigues M. L. Coelho C. (2019). The still underestimated problem of fungal diseases worldwide. *Front. Microbiol.* 10:214. 10.3389/FMICB.2019.00214 30809213 PMC6379264

[B9] Anwar M. A. Shah M. Kim J. Choi S. (2019). Recent clinical trends in toll-like receptor targeting therapeutics. *Med. Res. Rev.* 39 1053–1090. 10.1002/MED.21553 30450666 PMC6587958

[B10] Arleevskaya M. I. Larionova R. V. Brooks W. H. Bettacchioli E. Renaudineau Y. (2019). Toll-like receptors, infections, and rheumatoid arthritis. *Clin. Rev. Allergy Immunol* 58 172–181. 10.1007/S12016-019-08742-Z 31144208

[B11] Avcilar H. Eken A. (2020). Could imiquimod (Aldara 5% cream) or other TLR7 agonists be used in the treatment of COVID-19? *Med. Hypotheses* 144:110202. 10.1016/J.MEHY.2020.110202 33254510 PMC7434307

[B12] Azar D. F. Haas M. Fedosyuk S. Rahaman M. H. Hedger A. Kobe B. (2020). Vaccinia virus immunomodulator A46: destructive interactions with MAL and MyD88 Shown by negative-stain electron microscopy. *Structure* 28 1271–1287.e5. 10.1016/j.str.2020.09.007 33035450

[B13] Barrat F. J. (2018). TLR8: no gain, no pain. *J. Exp. Med.* 215:2964. 10.1084/JEM.20181899 30455266 PMC6279395

[B14] Barratt-Due A. Pischke S. E. Nilsson P. H. Espevik T. Mollnes T. E. (2017). Dual inhibition of complement and Toll-like receptors as a novel approach to treat inflammatory diseases—C3 or C5 emerge together with CD14 as promising targets. *J. Leukoc. Biol.* 101 193–204. 10.1189/JLB.3VMR0316-132R 27581539 PMC5166441

[B15] Baskar S. Klein A. L. Zeft A. (2016). The use of IL-1 receptor antagonist (Anakinra) in idiopathic recurrent pericarditis: a narrative review. *Cardiol. Res. Pract.* 2016:7840724. 10.1155/2016/7840724 26942035 PMC4752980

[B16] Bezemer G. F. G. Garssen J. (2021). TLR9 and COVID-19: a multidisciplinary theory of a multifaceted therapeutic target. *Front. Pharmacol.* 11:601685. 10.3389/FPHAR.2020.601685 33519463 PMC7844586

[B17] Boehme K. W. Guerrero M. Compton T. (2006). Human cytomegalovirus envelope glycoproteins B and H are necessary for TLR2 Activation in permissive cells. *J. Immunol.* 177 7094–7102. 10.4049/jimmunol.177.10.7094 17082626

[B18] Bourgeois C. Kuchler K. (2012). Fungal pathogens—a sweet and sour treat for toll-like receptors. *Front. Cell. Infect. Microbiol.* 2:142. 10.3389/FCIMB.2012.00142 23189270 PMC3504294

[B19] Braunstein M. J. Kucharczyk J. Adams S. (2018). Targeting Toll-like receptors for cancer therapy. *Target. Oncol.* 13 583–598. 10.1007/s11523-018-0589-7 30229471

[B20] Brown G. C. (2019). The endotoxin hypothesis of neurodegeneration. *J. Neuroinflammation* 161 1–10. 10.1186/S12974-019-1564-7 31519175 PMC6744684

[B21] Bruno K. Woller S. A. Miller Y. I. Yaksh T. L. Wallace M. Beaton G. (2018). Targeting Toll-like receptor-4 (TLR4) – emerging therapeutic target for persistent pain states. *Pain* 159:1908. 10.1097/J.PAIN.0000000000001306 29889119 PMC7890571

[B22] Burness C. B. McKeage K. (2015). Adalimumab: a review in chronic plaque psoriasis. *Drugs* 75 2119–2130. 10.1007/s40265-015-0503-x 26586242

[B23] Cao P. Luo W.-W. Li C. Tong Z. Zheng Z.-Q. Zhou L. (2019). The heterogeneous nuclear ribonucleoprotein hnRNPM inhibits RNA virus-triggered innate immunity by antagonizing RNA sensing of RIG-I-like receptors. *PLoS Pathog.* 15:e1007983. 10.1371/JOURNAL.PPAT.1007983 31433824 PMC6703689

[B24] Carlson R. D. Flickinger J. C. Jr. Snook A. E. (2020). Talkin’ toxins: from coley’s to modern cancer immunotherapy. *Toxins (Basel)* 12:241. 10.3390/TOXINS12040241 32283684 PMC7232517

[B25] Casilag F. Franck S. Matarazzo L. Figeac M. Michelet R. Kloft C. (2020). Boosting toll-like receptor 4 signaling enhances the therapeutic outcome of antibiotic therapy in pneumococcal pneumonia. *bioRxiv* [Preprint] 10.1101/2020.02.18.955500 34260199

[B26] Casilag F. Matarazzo L. Franck S. Figeac M. Michelet R. Kloft C. (2021). The biosynthetic monophosphoryl lipid a enhances the therapeutic outcome of antibiotic therapy in pneumococcal pneumonia. *ACS Infect. Dis.* 7 2164–2175. 10.1021/ACSINFECDIS.1C00176 34260199

[B27] Chen R. Alvero A. B. Silasi D. A. Steffensen K. D. Mor G. (2008). Cancers take their toll - the function and regulation of toll-like receptors in cancer cells. *Oncogene* 27 225–233. 10.1038/sj.onc.1210907 18176604

[B28] Cheng Z. Taylor B. Ourthiague D. R. Hoffmann A. (2015). Distinct single-cell signaling characteristics are conferred by the MyD88 and TRIF pathways during TLR4 activation. *Sci. Signal.* 8:ra69. 10.1126/SCISIGNAL.AAA5208 26175492 PMC6764925

[B29] Choudhury A. Mukherjee S. (2020). In silico studies on the comparative characterization of the interactions of SARS-CoV-2 spike glycoprotein with ACE-2 receptor homologs and human TLRs. *J. Med. Virol.* 92 2105–2113. 10.1002/JMV.25987 32383269 PMC7267663

[B30] Christou L. (2011). The global burden of bacterial and viral zoonotic infections. *Clin. Microbiol. Infect.* 17 326–330. 10.1111/J.1469-0691.2010.03441.X 21129102 PMC7129620

[B31] Cognasse F. Nguyen K. A. Damien P. McNicol A. Pozzetto B. Hamzeh-Cognasse H. (2015). The inflammatory role of platelets via their TLRs and siglec receptors. *Front. Immunol.* 6:83. 10.3389/fimmu.2015.00083 25784910 PMC4345914

[B32] de Oliviera Nascimento L. Massari P. Wetzler L. M. (2012). The role of TLR2 in infection and immunity. *Front. Immunol.* 3:79. 10.3389/FIMMU.2012.00079 22566960 PMC3342043

[B33] El-Zayat S. R. Sibaii H. Mannaa F. A. (2019). Toll-like receptors activation, signaling, and targeting: an overview. *Bull. Natl. Res. Cent.* 43:187. 10.1186/S42269-019-0227-2

[B34] Firestein G. S. McInnes I. B. (2017). Immunopathogenesis of rheumatoid arthritis. *Immunity* 46 183–196. 10.1016/j.immuni.2017.02.006 28228278 PMC5385708

[B35] Fitzgerald K. A. Kagan J. C. (2020). Toll-like Receptors and the control of immunity. *Cell* 180 1044–1066. 10.1016/J.CELL.2020.02.041 32164908 PMC9358771

[B36] Gabarin R. S. Li M. Zimmel P. A. Marshall J. C. Li Y. Zhang H. (2021). Intracellular and extracellular lipopolysaccharide signaling in sepsis: avenues for novel therapeutic strategies. *J. Innate Immun.* 18 1–10. 10.1159/000515740 34004605 PMC8613564

[B37] Gäbele E. Mühlbauer M. Dorn C. Weiss T. S. Froh M. Schnabl B. (2008). Role of TLR9 in hepatic stellate cells and experimental liver fibrosis. *Biochem. Biophys. Res. Commun.* 376 271–276. 10.1016/J.BBRC.2008.08.096 18760996

[B38] Gadanec L. K. McSweeney K. R. Qaradakhi T. Ali B. Zulli A. Apostolopoulos V. (2021). Can SARS-CoV-2 virus use multiple receptors to enter host cells? *Int. J. Mol. Sci.* 22 1–35. 10.3390/ijms22030992 33498183 PMC7863934

[B39] Gay N. J. (2019). Role of self-organising myddosome oligomers in inflammatory signalling by toll-like receptors. *BMC Biol.* 17:15. 10.1186/S12915-019-0637-5 30786893 PMC6383289

[B40] Goh F. G. Midwood K. S. (2012). Intrinsic danger: activation of toll-like receptors in rheumatoid arthritis. *Rheumatology* 51 7–23. 10.1093/rheumatology/ker257 21984766

[B41] Hammerich L. Marron T. U. Upadhyay R. Svensson-Arvelund J. Dhainaut M. Hussein S. (2019). Systemic clinical tumor regressions and potentiation of PD1 blockade with in situ vaccination. *Nat. Med.* 255 814–824. 10.1038/s41591-019-0410-x 30962585

[B42] Harte M. T. Haga I. R. Maloney G. Gray P. Reading P. C. Bartlett N. W. (2003). The poxvirus protein A52R targets toll-like receptor signaling complexes to suppress host defense. *J. Exp. Med.* 197 343–351. 10.1084/jem.20021652 12566418 PMC2193841

[B43] He L. Han G. Wu S. Du S. Zhang Y. Liu W. (2020). Toll-like receptor 7 contributes to neuropathic pain by activating NF-κB in primary sensory neurons. *Brain. Behav. Immun.* 87 840–851. 10.1016/J.BBI.2020.03.019 32205121 PMC7316623

[B44] Hedayat M. Netea M. G. Rezaei N. (2011). Targeting of toll-like receptors: a decade of progress in combating infectious diseases. *Lancet Infect. Dis.* 11 702–712. 10.1016/S1473-3099(11)70099-8 21719349

[B45] Hiraku Y. Kawanishi S. Ohshima H. (2014). *Cancer and Inflammation Mechanisms: Chemical, Biological, and Clinical Aspects.* Hoboken, NJ: John Wiley & Sons, Inc. 10.1002/9781118826621

[B46] Hosseini A. M. Majidi J. Baradaran B. Yousefi M. (2015). Toll-like receptors in the pathogenesis of autoimmune diseases. *Adv. Pharm. Bull.* 5 605–614. 10.15171/apb.2015.082 26793605 PMC4708030

[B47] Huang B. Zhao J. Unkeless J. C. Feng Z. H. Xiong H. (2008). TLR signaling by tumor and immune cells: a double-edged sword. *Oncogene* 27 218–224. 10.1038/sj.onc.1210904 18176603

[B48] Huang L. Xu H. Peng G. (2018). TLR-mediated metabolic reprogramming in the tumor microenvironment: potential novel strategies for cancer immunotherapy. *Cell. Mol. Immunol.* 15 428–437. 10.1038/cmi.2018.4 29553135 PMC6068099

[B49] Israël A. (2010). The IKK complex, a central regulator of NF-κB activation. *Cold Spring Harb. Perspect. Biol.* 2:a000158. 10.1101/CSHPERSPECT.A000158 20300203 PMC2829958

[B50] Jurga A. M. Rojewska E. Piotrowska A. Makuch W. Pilat D. Przewlocka B. (2016). Blockade of toll-like receptors (TLR2, TLR4) attenuates pain and potentiates buprenorphine analgesia in a rat neuropathic pain model. *Neural Plast.* 2016:5238730. 10.1155/2016/5238730 26962463 PMC4709736

[B51] Kaczanowska S. Joseph A. M. Davila E. (2013). TLR agonists: our best frenemy in cancer immunotherapy. *J. Leukoc. Biol.* 93 847–863. 10.1189/JLB.1012501 23475577 PMC3656332

[B52] Kaushik D. Bhandari R. Kuhad A. (2021). TLR4 as a therapeutic target for respiratory and neurological complications of SARS-CoV-2. *Expert Opin. Ther. Targets* 25 491–508. 10.1080/14728222.2021.1918103 33857397 PMC8095161

[B53] Kawai T. Akira S. (2009). The roles of TLRs, RLRs and NLRs in pathogen recognition. *Int. Immunol.* 21 317–337. 10.1093/INTIMM/DXP017 19246554 PMC2721684

[B54] Kawai T. Akira S. (2011). Toll-like receptors and their crosstalk with other innate receptors in infection and immunity. *Immunity* 34 637–650. 10.1016/j.immuni.2011.05.006 21616434

[B55] Kawasaki T. Kawai T. (2014). Toll-like receptor signaling pathways. *Front. Immunol.* 5:461. 10.3389/FIMMU.2014.00461 25309543 PMC4174766

[B56] Knapp S. (2010). Update on the role of toll-like receptors during bacterial infections and sepsis. *Wien. Med. Wochenschr.* 160 107–111. 10.1007/S10354-010-0765-6 20364412

[B57] Korman N. J. (2020). Management of psoriasis as a systemic disease: what is the evidence? *Br. J. Dermatol.* 182 840–848. 10.1111/bjd.18245 31225638 PMC7187293

[B58] Kuhn A. Luger T. A. (2010). Psoriasis: is ustekinumab superior to etanercept for psoriasis? *Nat. Rev. Rheumatol.* 6 500–501. 10.1038/nrrheum.2010.134 20808304

[B59] Kumar S. Sunagar R. Gosselin E. (2019). Bacterial protein toll-like-receptor agonists: a novel perspective on vaccine adjuvants. *Front. Immunol.* 10:1144. 10.3389/FIMMU.2019.01144 31191528 PMC6549121

[B60] Kuzmich N. N. Sivak K. V. Chubarev V. N. Porozov Y. B. Savateeva-Lyubimova T. N. Peri F. (2017). TLR4 signaling pathway modulators as potential therapeutics in inflammation and sepsis. *Vaccines* 5:34. 10.3390/VACCINES5040034 28976923 PMC5748601

[B61] Lai C. Y. Su Y. W. Lin K. I. Hsu L. C. Chuang T. H. (2017). Natural modulators of endosomal toll-like receptor-mediated psoriatic skin inflammation. *J. Immunol. Res.* 2017:7807313. 10.1155/2017/7807313 28894754 PMC5574364

[B62] Landais I. Pelton C. Streblow D. DeFilippis V. McWeeney S. Nelson J. A. (2015). Human cytomegalovirus miR-UL112-3p Targets TLR2 and modulates the TLR2/IRAK1/NFκB signaling pathway. *PLoS Pathog.* 11:e1004881. 10.1371/JOURNAL.PPAT.1004881 25955717 PMC4425655

[B63] Larue H. Ayari C. Bergeron A. Fradet Y. (2013). Toll-like receptors in urothelial cells – targets for cancer immunotherapy. *Nat. Rev. Urol.* 10 537–545. 10.1038/nrurol.2013.153 23979666

[B64] Leonard J. N. Ghirlando R. Askins J. Bell J. K. Margulies D. H. Davies D. R. (2008). *The TLR3 Signaling Complex Forms by Cooperative Receptor Dimerization.* Available online at: www.pnas.org/cgi/content/full/ (accessed March 4, 2021) 10.1073/pnas.0710779105 PMC2224197 18172197

[B65] Leonardi C. Papp K. Strober B. Reich K. Asahina A. Gu Y. (2011). The long-term safety of adalimumab treatment in moderate to severe psoriasis: a comprehensive analysis of all adalimumab exposure in all clinical trials. *Am. J. Clin. Dermatol.* 12 321–337. 10.2165/11587890-000000000-00000 21834597

[B66] Leonardi C. Papp K. Strober B. Thaçi D. Warren R. B. Tyring S. (2019). Comprehensive long-term safety of adalimumab from 18 clinical trials in adult patients with moderate-to-severe plaque psoriasis. *Br. J. Dermatol.* 180 76–85. 10.1111/bjd.17084 30169904

[B67] Lester S. N. Li K. (2014). Toll-like receptors in antiviral innate immunity. *J. Mol. Biol.* 426 1246–1264. 10.1016/j.jmb.2013.11.024 24316048 PMC3943763

[B68] Li E. Zhang Y. (2014). DNA methylation in mammals. *Cold Spring Harb. Perspect. Biol.* 6 19133–19134. 10.1101/cshperspect.a019133 24789823 PMC3996472

[B69] Li H. Sun H. Xu Y. Xing G. Wang X. (2021). Curcumin plays a protective role against septic acute kidney injury by regulating the TLR9 signaling pathway. *Transl. Androl. Urol.* 10:2103. 10.21037/TAU-21-385 34159091 PMC8185681

[B70] Li Z. Cai S. Sun Y. Li L. Ding S. Wang X. (2020). When STING meets viruses: sensing, trafficking and response. *Front. Immunol.* 11:2064. 10.3389/FIMMU.2020.02064 33133062 PMC7550420

[B71] Lionakis M. S. Levitz S. M. (2018). Host control of fungal infections: lessons from basic studies and human cohorts. *Annu. Rev. Immunol.* 36 157–191. 10.1146/annurev-immunol-042617-053318 29237128

[B72] Liu F. Li X. Yue H. Ji J. You M. Ding L. (2017). TLR-induced SMPD3 defects enhance inflammatory response of B cell and macrophage in the pathogenesis of SLE. *Scand. J. Immunol.* 86 377–388. 10.1111/sji.12611 28889482

[B73] Mao C. Gorbet M.-J. Singh A. Ranjan A. Fiering S. (2020). In situ vaccination with nanoparticles for cancer immunotherapy: understanding the immunology. *Int. J. Hyperthermia* 37:4. 10.1080/02656736.2020.1810333 33455477 PMC8189648

[B74] Matsushima N. Tanaka T. Enkhbayar P. Mikami T. Taga M. Yamada K. (2007). Comparative sequence analysis of leucine-rich repeats (LRRs) within vertebrate toll-like receptors. *BMC Genomics* 8:124. 10.1186/1471-2164-8-124 17517123 PMC1899181

[B75] McGettrick A. F. O’Neill L. A. (2010). Localisation and trafficking of Toll-like receptors: an important mode of regulation. *Curr. Opin. Immunol.* 22 20–27. 10.1016/J.COI.2009.12.002 20060278

[B76] McInnes I. B. Schett G. (2017). Pathogenetic insights from the treatment of rheumatoid arthritis. *Lancet* 389 2328–2337. 10.1016/S0140-6736(17)31472-1 28612747

[B77] Moghadam S. O. Nowroozi M. R. (2019). Toll-like receptors: the role in bladder cancer development, progression and immunotherapy. *Scand. J. Immunol.* 90:e12818. 10.1111/SJI.12818 31448424

[B78] Monnet E. Choy E. H. McInnes I. Kobakhidze T. de Graaf K. Jacqmin P. (2020). Efficacy and safety of NI-0101, an anti-toll-like receptor 4 monoclonal antibody, in patients with rheumatoid arthritis after inadequate response to methotrexate: a phase II study. *Ann. Rheum. Dis.* 79 316–323. 10.1136/ANNRHEUMDIS-2019-216487 31892533

[B79] Muccioli M. Benencia F. (2014). Toll-like receptors in ovarian cancer as targets for immunotherapies. *Front. Immunol.* 5:341. 10.3389/FIMMU.2014.00341 25101083 PMC4105689

[B80] Murad Y. M. Clay T. M. (2009). CpG oligodeoxynucleotides as TLR9 agonists: therapeutic applications in cancer. *BioDrugs* 23 361–375. 10.2165/11316930-000000000-00000 19894778

[B81] Nazmi A. Mukherjee S. Kundu K. Dutta K. Mahadevan A. Shankar S. K. (2014). TLR7 is a key regulator of innate immunity against Japanese encephalitis virus infection. *Neurobiol. Dis.* 69 235–247. 10.1016/j.nbd.2014.05.036 24909816

[B82] Netea M. G. Van derMeer J. W. M. Kullberg B. J. (2007). “Recognition of fungal pathogens by Toll-like receptors,” in *Immunology of Fungal Infections*, eds Brown G. D. Netea M. G. (Dordrecht: Springer), 259–272. 10.1007/1-4020-5492-0_11

[B83] Nie L. Cai S.-Y. Shao J.-Z. Chen J. (2018). Toll-like receptors, associated biological roles, and signaling networks in non-mammals. *Front. Immunol.* 9:1523. 10.3389/FIMMU.2018.01523 30034391 PMC6043800

[B84] Nijland R. Hofland T. van Strijp J. A. (2014). Recognition of LPS by TLR4: potential for anti-inflammatory therapies. *Mar. Drugs* 12 4260–4273. 10.3390/MD12074260 25056632 PMC4113827

[B85] O’Neill L. A. J. Bowie A. G. (2007). The family of five: TIR-domain-containing adaptors in toll-like receptor signalling. *Nat. Rev. Immunol.* 7 353–364. 10.1038/nri2079 17457343

[B86] O’Neill L. A. J. Golenbock D. Bowie A. G. (2013). The history of toll-like receptors-redefining innate immunity. *Nat. Rev. Immunol.* 13 453–460. 10.1038/nri3446 23681101

[B87] Ono Y. Maejima Y. Saito M. Sakamoto K. Horita S. Shimomura K. (2020). TAK-242, a specific inhibitor of Toll-like receptor 4 signalling, prevents endotoxemia-induced skeletal muscle wasting in mice. *Sci. Rep.* 10 1–13. 10.1038/s41598-020-57714-3 31959927 PMC6970997

[B88] Opal S. M. Laterre P.-F. Francois B. LaRosa S. P. Angus D. C. Mira J.-P. (2013). Effect of eritoran, an antagonist of MD2-TLR4, on mortality in patients with severe sepsis: the ACCESS randomized trial. *JAMA* 309 1154–1162. 10.1001/JAMA.2013.2194 23512062

[B89] Ospelt C. Brentano F. Rengel Y. Stanczyk J. Kolling C. Tak P. P. (2008). Overexpression of toll-like receptors 3 and 4 in synovial tissue from patients with early rheumatoid arthritis: toll-like receptor expression in early and longstanding arthritis. *Arthritis Rheum.* 58 3684–3692. 10.1002/art.24140 19035519

[B90] Ospelt C. Gay S. (2010). TLRs and chronic inflammation. *Int. J. Biochem. Cell Biol.* 42 495–505. 10.1016/j.biocel.2009.10.010 19840864

[B91] Pantazi I. Al-Qahtani A. A. Alhamlan F. S. Alothaid H. Matou-Nasri S. Sourvinos G. (2021). SARS-CoV-2/ACE2 interaction suppresses IRAK-M expression and promotes pro-inflammatory cytokine production in macrophages. *Front. Immunol.* 12:683800. 10.3389/FIMMU.2021.683800 34248968 PMC8261299

[B92] Park B. S. Lee J.-O. (2013). Recognition of lipopolysaccharide pattern by TLR4 complexes. *Exp. Mol. Med.* 45:e66. 10.1038/EMM.2013.97 24310172 PMC3880462

[B93] Parker D. Prince A. (2012). *Staphylococcus aureus* induces type I IFN signaling in dendritic cells via TLR9. *J. Immunol.* 189 4040–4046. 10.4049/jimmunol.1201055 22962685 PMC3466375

[B94] Patel M. C. Shirey K. A. Pletneva L. M. Boukhvalova M. S. Garzino-Demo A. Vogel S. N. (2014). Novel drugs targeting toll-like receptors for antiviral therapy. *Future Virol.* 9 811–829. 10.2217/fvl.14.70 25620999 PMC4303062

[B95] Perales-Linares R. Navas-Martin S. (2013). Toll-like receptor 3 in viral pathogenesis: friend or foe? *Immunology* 140 153–167. 10.1111/imm.12143 23909285 PMC3784162

[B96] Pierer M. Wagner U. Rossol M. Ibrahim S. (2011). Toll-like receptor 4 is involved in inflammatory and joint destructive pathways in collagen-induced arthritis in DBA1J mice. *PLoS One* 6:e23539. 10.1371/JOURNAL.PONE.0023539 21858160 PMC3157404

[B97] Płóciennikowska A. Hromada-Judycka A. Borzęcka K. Kwiatkowska K. (2015). Co-operation of TLR4 and raft proteins in LPS-induced pro-inflammatory signaling. *Cell. Mol. Life Sci.* 72 557–581. 10.1007/S00018-014-1762-5 25332099 PMC4293489

[B98] Pradere J. P. Dapito D. H. Schwabe R. F. (2014). The Yin and Yang of toll-like receptors in cancer. *Oncogene* 33 3485–3495. 10.1038/onc.2013.302 23934186 PMC4059777

[B99] Proud P. C. Tsitoura D. Watson R. J. Chua B. Y. Aram M. J. Bewley K. R. (2021). Prophylactic intranasal administration of a TLR2/6 agonist reduces upper respiratory tract viral shedding in a SARS-CoV-2 challenge ferret model. *EBioMedicine* 63:103153. 10.1016/J.EBIOM.2020.103153 33279857 PMC7711201

[B100] Rahmani F. Rezaei N. (2016). Therapeutic targeting of toll-like receptors: a review of toll-like receptors and their signaling pathways in psoriasis. *Expert Rev. Clin. Immunol.* 12 1289–1298. 10.1080/1744666X.2016.1204232 27359083

[B101] Rakoff-Nahoum S. Medzhitov R. (2009). Toll-like receptors and cancer. *Nat. Rev. Cancer* 9 57–63. 10.1038/nrc2541 19052556

[B102] Rehwinkel J. Gack M. U. (2020). RIG-I-like receptors: their regulation and roles in RNA sensing. *Nat. Rev. Immunol.* 201 537–551. 10.1038/s41577-020-0288-3 32203325 PMC7094958

[B103] Ren V. Dao H. (2013). Potential role of ixekizumab in the treatment of moderate-to-severe plaque psoriasis. *Clin. Cosmet. Investig. Dermatol.* 6 75–80. 10.2147/CCID.S42424 23515267 PMC3600940

[B104] Romani L. (2011). Immunity to fungal infections. *Nat. Rev. Immunol.* 11 275–288. 10.1038/nri2939 21394104

[B105] Sacre S. M. Lo A. Gregory B. Simmonds R. E. Williams L. Feldmann M. (2008). Inhibitors of TLR8 reduce TNF production from human rheumatoid synovial membrane cultures. *J. Immunol.* 181 8002–8009. 10.4049/jimmunol.181.11.8002 19017992

[B106] Salvi V. Nguyen H. O. Sozio F. Schioppa T. Laffranchi M. Scapini P. (2021). SARS-CoV-2-associated ssRNAs activate inflammation and immunity via TLR7/8. *bioRxiv* [Preprint] 10.1101/2021.04.15.439839 2021.04.15.439839, PMC8492321 34375313

[B107] Samarpita S. Kim J. Y. Rasool M. K. Kim K. S. (2020). Investigation of toll-like receptor (TLR) 4 inhibitor TAK-242 as a new potential anti-rheumatoid arthritis drug. *Arthritis Res. Ther.* 22 1–10. 10.1186/s13075-020-2097-2 31973752 PMC6979396

[B108] Savva A. Roger T. (2013). Targeting toll-like receptors: promising therapeutic strategies for the management of sepsis-associated pathology and infectious diseases. *Front. Immunol.* 4:387. 10.3389/FIMMU.2013.00387 24302927 PMC3831162

[B109] Scho M. Scho M. (2008). TLR7 and TLR8 as targets in cancer therapy. *Oncogene* 27 190–199. 10.1038/sj.onc.1210913 18176600

[B110] Shah M. Choi S. (2017). Toll-like receptor-dependent negative effects of opioids: a battle between analgesia and hyperalgesia. *Front. Immunol.* 8:642. 10.3389/FIMMU.2017.00642 28620391 PMC5450035

[B111] Shear M. J. Turner F. C. Perrault A. Shovelton T. (1943). Chemical treatment of tumors. V. isolation of the hemorrhage-producing fraction from *Serratia marcescens* (*Bacillus prodigiosus*) culture filtrate. *JNCI J. Natl. Cancer Inst.* 4 81–97. 10.1093/JNCI/4.1.81

[B112] Shekarian T. Valsesia-Wittmann S. Brody J. Michallet M. C. Depil S. Caux C. (2017). Pattern recognition receptors: immune targets to enhance cancer immunotherapy. *Ann. Oncol.* 28 1756–1766. 10.1093/annonc/mdx179 28444111

[B113] Shi M. Chen X. Ye K. Yao Y. Li Y. (2016). Application potential of toll-like receptors in cancer immunotherapy: systematic review. *Medicine (Baltimore)* 95:e3951. 10.1097/MD.0000000000003951 27336891 PMC4998329

[B114] Simó R. Stitt A. W. Gardner T. W. (2018). Neurodegeneration in diabetic retinopathy: does it really matter? *Diabetol* 61 1902–1912. 10.1007/S00125-018-4692-1 30030554 PMC6096638

[B115] Smatti M. K. Cyprian F. S. Nasrallah G. K. Al Thani A. A. Almishal R. O. Yassine H. M. (2019). Viruses and autoimmunity: a review on the potential interaction and molecular mechanisms. *Viruses* 11:762. 10.3390/v11080762 31430946 PMC6723519

[B116] Stack J. Haga I. R. Schröder M. Bartlett N. W. Maloney G. Reading P. C. (2005). Vaccinia virus protein A46R targets multiple toll-like-interleukin-1 receptor adaptors and contributes to virulence. *J. Exp. Med.* 201 1007–1018. 10.1084/jem.20041442 15767367 PMC2213104

[B117] Sweeney C. M. Tobin A. M. Kirby B. (2011). Innate immunity in the pathogenesis of psoriasis. *Arch. Dermatol. Res.* 303 691–705. 10.1007/s00403-011-1169-1 21863252

[B118] Szajnik M. Szczepanski M. J. Czystowska M. Elishaev E. Mandapathil M. Nowak-Markwitz E. (2009). TLR4 signaling induced by lipopolysaccharide or paclitaxel regulates tumor survival and chemoresistance in ovarian cancer. *Oncogene* 28 4353–4363. 10.1038/onc.2009.289 19826413 PMC2794996

[B119] Takeda K. Akira S. (2015). Toll-like receptors. *Curr. Protoc. Immunol.* 109 14.12.1–14.12.10. 10.1002/0471142735.im1412s109 25845562

[B120] Takeuchi O. Akira S. (2007). Recognition of viruses by innate immunity. *Immunol. Rev.* 220 214–224. 10.1111/j.1600-065X.2007.00562.x 17979849

[B121] Takeuchi O. Akira S. (2010). Pattern recognition receptors and inflammation. *Cell* 140 805–820. 10.1016/j.cell.2010.01.022 20303872

[B122] Thakur K. K. Saini J. Mahajan K. Singh D. Jayswal D. P. Mishra S. (2017). Therapeutic implications of toll-like receptors in peripheral neuropathic pain. *Pharmacol. Res.* 115 224–232. 10.1016/J.PHRS.2016.11.019 27894923

[B123] Theofilopoulos A. N. Kono D. H. Baccala R. (2017). The multiple pathways to autoimmunity. *Nat. Immunol.* 18 716–724. 10.1038/ni.3731 28632714 PMC5791156

[B124] Thompson J. M. Iwasaki A. (2008). Toll-like receptors regulation of viral infection and disease. *Adv. Drug Deliv. Rev.* 60 786–794. 10.1016/j.addr.2007.11.003 18280610 PMC2410298

[B125] Toubi E. Vadasz Z. (2019). Innate immune-responses and their role in driving autoimmunity. *Autoimmun. Rev.* 18 306–311. 10.1016/j.autrev.2018.10.005 30639645

[B126] Uehata T. Takeuchi O. (2020). RNA recognition and immunity—innate immune sensing and its posttranscriptional regulation mechanisms. *Cells* 9:1701. 10.3390/CELLS9071701 32708595 PMC7407594

[B127] Urban-Wojciuk Z. Khan M. M. Oyler B. L. Fåhraeus R. Marek-Trzonkowska N. Nita-Lazar A. (2019). The role of TLRs in anti-cancer immunity and tumor rejection. *Front. Immunol.* 10:2388. 10.3389/FIMMU.2019.02388 31695691 PMC6817561

[B128] Van Der Graaf C. A. A. Netea M. G. Verschueren I. Van Der Meer J. W. M. Kullberg B. J. (2005). Differential cytokine production and toll-like receptor signaling pathways by Candida albicans blastoconidia and hyphae. *Infect. Immun.* 73 7458–7464. 10.1128/IAI.73.11.7458-7464.2005 16239547 PMC1273874

[B129] Ve T. Vajjhala P. R. Hedger A. Croll T. Dimaio F. Horsefield S. (2017). Structural basis of TIR-domain-assembly formation in MAL- and MyD88-dependent TLR4 signaling. *Nat. Struct. Mol. Biol.* 24 743–751. 10.1038/nsmb.3444 28759049 PMC8059215

[B130] Vijay K. (2018). Toll-like receptors in immunity and inflammatory diseases: past, present, and future. *Int. Immunopharmacol.* 59 391–412. 10.1016/J.INTIMP.2018.03.002 29730580 PMC7106078

[B131] Wagner H. (2004). The immunobiology of the TLR9 subfamily. *Trends Immunol.* 25 381–386. 10.1016/J.IT.2004.04.011 15207506

[B132] White J. M. Whittaker G. R. (2016). Fusion of enveloped viruses in endosomes. *Traffic* 17:593. 10.1111/TRA.12389 26935856 PMC4866878

[B133] Wu K. Zhang H. Fu Y. Zhu Y. Kong L. Chen L. (2018). TLR4/MyD88 signaling determines the metastatic potential of breast cancer cells. *Mol. Med. Rep.* 18:3411. 10.3892/MMR.2018.9326 30066873 PMC6102647

[B134] Yang X. Cheng Y. Li C. (2017). The role of TLRs in cervical cancer with HPV infection: a review. *Signal Transduct. Target. Ther.* 2 1–10. 10.1038/sigtrans.2017.55 29263932 PMC5668671

[B135] Yang Y. Lv J. Jiang S. Ma Z. Wang D. Hu W. (2016). The emerging role of toll-like receptor 4 in myocardial inflammation. *Cell Death Dis.* 7:e2234. 10.1038/CDDIS.2016.140 27228349 PMC4917669

[B136] Yu X. Chen R. Wang F. Liu W. Zhang W. Gong M. (2021). Pattern recognition receptor-initiated innate immune responses in mouse prostatic epithelial cells. *Biol. Reprod.* 105 113–127. 10.1093/BIOLRE/IOAB076 33899078

[B137] Zamora-Atenza C. Diaz-Torne C. Geli C. Diaz-Lopez C. Ortiz M. A. Moya P. (2014). Adalimumab regulates intracellular TNFα production in patients with rheumatoid arthritis. *Arthritis Res. Ther.* 16:R153. 10.1186/AR4615 25037855 PMC4223509

[B138] Zhang J. Yu C. Zhang X. Chen H. Dong J. Lu W. (2018). Porphyromonas gingivalis lipopolysaccharide induces cognitive dysfunction, mediated by neuronal inflammation via activation of the TLR4 signaling pathway in C57BL/6 mice. *J. Neuroinflammation* 15 1–14. 10.1186/S12974-017-1052-X 29426327 PMC5810193

[B139] Zhang Y. Li Y. Li Y. Li R. Ma Y. Wang H. (2015). Chloroquine inhibits MGC803 gastric cancer cell migration via the toll-like receptor 9/nuclear factor kappa B signaling pathway. *Mol. Med. Rep.* 11 1366–1371. 10.3892/MMR.2014.2839 25369757

[B140] Zhao Y. Karijolich J. (2019). Know thyself: RIG-I-like receptor sensing of DNA virus infection. *J. Virol.* 93:e01085-19. 10.1128/JVI.01085-19 31511389 PMC6854496

[B141] Zheng M. Karki R. Williams E. P. Yang D. Fitzpatrick E. Vogel P. (2021). TLR2 senses the SARS-CoV-2 envelope protein to produce inflammatory cytokines. *Nat. Immunol.* 227 829–838. 10.1038/s41590-021-00937-x 33963333 PMC8882317

[B142] Zweegers J. Groenewoud J. M. M. van den Reek J. M. P. A. Otero M. E. van de Kerkhof P. C. M. Driessen R. J. B. (2017). Comparison of the 1- and 5-year effectiveness of adalimumab, etanercept and ustekinumab in patients with psoriasis in daily clinical practice: results from the prospective BioCAPTURE registry. *Br. J. Dermatol.* 176 1001–1009. 10.1111/bjd.15023 27579864

